# Prevention of elastase-induced emphysema in placenta growth factor knock-out mice

**DOI:** 10.1186/1465-9921-10-115

**Published:** 2009-11-23

**Authors:** Shih Lung Cheng, Hao Chien Wang, Chong Jen Yu, Po Nien Tsao, Peter Carmeliet, Shi Jung Cheng, Pan Chyr Yang

**Affiliations:** 1 Department of Internal Medicine, Far Eastern Memorial Hospital, Taiwan; 2 Department of Chemical Engineering and Materials Science, Yuan-Ze University, Taiwan; 3 Department of Internal Medicine, National Taiwan University Hospital, Taiwan; 4 Department of Pediatrics, National Taiwan University Hospital, Taiwan; 5 Vesalius Research Center, VIB, 3000 Leuven, Belgium; 6 Vesalius Research Center, K.U. Leuven, 3000 Leuven, Belgium; 7 Division of Oral and Maxillofacial Surgery, Department of Dentistry, National Taiwan University Hospital, Taipei, Taiwan

## Abstract

**Background:**

Although both animal and human studies suggested the association between placenta growth factor (PlGF) and chronic obstructive pulmonary disease (COPD), especially lung emphysema, the role of PlGF in the pathogenesis of emphysema remains to be clarified. This study hypothesizes that blocking PlGF prevents the development of emphysema.

**Methods:**

Pulmonary emphysema was induced in PlGF knock-out (KO) and wild type (WT) mice by intra-tracheal instillation of porcine pancreatic elastase (PPE). A group of KO mice was then treated with exogenous PlGF and WT mice with neutralizing anti-VEGFR1 antibody. Tumor necrosis factor alpha (TNF-α), matrix metalloproteinase-9 (MMP-9), and VEGF were quantified. Apoptosis measurement and immuno-histochemical staining for VEGF R1 and R2 were performed in emphysematous lung tissues.

**Results:**

After 4 weeks of PPE instillation, lung airspaces enlarged more significantly in WT than in KO mice. The levels of TNF-α and MMP-9, but not VEGF, increased in the lungs of WT compared with those of KO mice. There was also increased in apoptosis of alveolar septal cells in WT mice. Instillation of exogenous PlGF in KO mice restored the emphysematous changes. The expression of both VEGF R1 and R2 decreased in the emphysematous lungs.

**Conclusion:**

In this animal model, pulmonary emphysema is prevented by depleting PlGF. When exogenous PlGF is administered to PlGF KO mice, emphysema re-develops, implying that PlGF contributes to the pathogenesis of emphysema.

## Background

Chronic obstructive pulmonary disease (COPD) affects over 18 million Americans and is the 4^th ^leading cause of death in the US. The disease burden will continue to increase globally as smoking rates climb in most developing countries [1]. Emphysema, a major component of COPD, is characterized by variable inflammatory cell infiltration, including neutrophils, alveolar macrophages, and CD4^+ ^and CD8^+ ^lymphocytes, as well as the presence of proteinase-anti-proteinase imbalance within the alveolar space, which leads to destruction and permanent enlargement of peripheral lung airspaces [2-6]. Pulmonary emphysema is defined as the abnormal enlargement of respiratory spaces with destruction of the alveolar walls. Experimental evidence supports the concept that proteases from activated macrophages and neutrophils degrade elastin and other structural proteins, thereby damaging alveolar units [5,7].

The "vascular hypothesis" of COPD is corroborated by a recent study showing that protein levels and messenger ribonucleic acid (mRNA) expression of both VEGF and its receptor are decreased in lung tissues of COPD patients [8]. Moreover, cigarette smoke disrupts components of the VEGF_165_-VEGFR2 and decreases the expression of VEGF and its receptors in the lungs of rats and humans [9]. Thus, VEGF signaling is considered mandatory for the maintenance of alveolar structures.

Placenta growth factor (PlGF) is an angiogenic growth factor, which is a 50-kDa glycosylated dimeric protein sharing 53% sequence homology at the amino acid level with VEGF [10]. Like VEGF, it exhibits mitogenic activity in cultured endothelial cells and induces angiogenesis *in vivo *[11]. PlGF mRNA is abundant in the placenta, thyroid, and lungs [12], but its biologic function in these tissues remains largely unclear. A previous study involving PlGF-transgenic mice demonstrates significantly enlarged air spaces and enhanced pulmonary compliance, a situation mimicking human pulmonary emphysema [13]. The increased PlGF expression was also shown in COPD patients [14].

Based on our previous results from transgenic mice and human subjects, it is postulated that PlGF may be involved in the inflammatory process related to emphysema. This study aimed to test this hypothesis by determining whether emphysema could be prevented in mice whose PlGF had been knocked out. It further aimed to elucidate the role of PlGF in the pathogenesis of emphysema.

## Methods

### Animals

The Animal Care and Use Committee of the National Taiwan University Hospital approved the following animal protocol. Breeding couples of wild-type (PlGF +/+), heterozygous type (PlGF +/-), and PlGF knock-out type (PlGF^-/-^) mice in a 50% 129Sv × 50% Swiss background were performed as described [15]. These mice were available from the Dr. Peter Carmeliet's animal lab. In breeding rooms, we maintained on a 12-hr light and dark cycle with constant temperature and humidity.

### Experimental animals and PPE-induced emphysema

The129/sw mice were anesthetized with intra-peritoneal urethane (120 mg/100 g) and given porcine pancreatic elastase (PPE) (Worthington; Biochem) at 4 mg/kg or saline (0.9% NaCl) alone via intra-tracheal instillation every week. These mice were then divided into 4 groups (n = 5 each), including the wild type (PlGF +/+, WT with PPE), heterozygous deficient (PlGF +/-, HE with PPE), homozygous deficient (PlGF -/-, KO with PPE), and control (PlGF +/+, PlGF +/- and PlGF -/- with saline; C, C+/- and C-/-, respectively). After 4 weeks of continuous treatment, the mice were sacrificed for study. After being anesthetized and exsanguinated, their lungs were inflated until visibly taut (maximum volume) with freshly prepared paraformaldehyde through tracheal cannula. The maximum volume was maintained for at least 2 minutes before the trachea was tied-off to maintain inflation. Two transverse tissue slabs were cut from the lungs and one from the right caudal lobe. The same locations were sampled in all mice. These tissues were embedded in paraffin and 4-μm serial sections were cut, individually handled and numbered, and transferred on to the slides.

Second, the PlGF KO mice were evaluated if they could re-develop emphysema. In these mice, PPE instillation followed by exogenous PlGF at 1 mg/kg dose via intra-tracheal route was done weekly. After 4 weeks, these KO mice were studies for measurement of airspace enlargement.

Third, the PlGF WT mice were instilled with PPE followed by VEGF R1 blocker agent (neutralizing antibody against mouse VEGF-R1, AF471; R&D Systems) at a dose of 10 μg/kg every week. After 4 weeks, these mice were sacrificed to measure emphysema development.

### Morphologic evaluation and quantification of emphysema

Quantitative histological measurements were made using an image analysis system, consisting of an Olympus CCD camera (Olympus, Tokyo, Japan). From each field, five areas of interest, free of airway and muscular blood vessels, were picked for measuring the number of intersections of virtual lines of known length with alveolar septa [16]. An increase in the average distance between Mean Linear Intercept (MLI) indicates enlarged airspaces. The areas of interest were also analyzed for tissue area and lung-air area. Volume density of the airspace (V _v(air, lung) _%) was also measured [17].

### Expression of inflammatory mediators and VEGF in bronchoalveolar lavage by ELISA

A 22-gauge cannula was inserted into the trachea, and both lungs were lavaged five times with 0.8 ml of PBS. The collected fluid was centrifuged at 400 × g for 10 minutes. The supernatant (bronchoalveolar lavage fluid) were divided into aliquots and stored at -80°C until analysis. The quantification for TNF-alpha, MMP-9 and VEGF were assayed by standardized sandwich enzyme-linked immunosorbent assay (ELISA) method (R&D Systems, Minneapolis, MN, USA) in duplicate according to the manufacturer's protocol.

### Western Blot analysis for TNF-alpha, MMP-9, VEGF, VEGFR1 and VEGFR2

Excised lungs were homogenized in a solution containing 1 mM EDTA, 0.5 mM aminoethylbenezenesulfonyl fluoride (AEBSF), 1 μg/ml Leupeptin, 1 μg/ml Aprotinin, 10 μg/ml Trypsin-Chymotrypsin inhibitor, 1 μg/ml Pepstatin A (all from Sigma). Proteins were resolved on 10% polyacrylamide gel and Western blots performed using standard techniques. Membranes were incubated overnight at 4°C with the following antibodies from Santa Cruz Biochemicals (Santa Cruz, CA): anti-MMP-9 diluted 1:500; anti-TNF-α diluted 1: 1000; anti-VEGF diluted 1: 1000; anti-VEGFR1 diluted 1: 1000; and anti-VEGFR2 diluted 1: 1000, respectively as the primary antibody, and a 1:600 dilution of anti-goat IgG-horseradish peroxidase (Santa Cruz, Cat # SC-2020) as the secondary antibody

### Quantification of apoptotic cell assay in emphysematous lungs

*In situ *nick end-labeling (TUNEL) was performed using the *in situ *cell death detection kit, Fluorescein (Roche, Applied Science. Cat. No.11684795910), which was also used for detecting and quantifying apoptosis (programmed cell death) at the single cell level, based on labeling of DNA strand breaks (TUNEL technology). Analysis was performed by fluorescence microscopy according to the manufacturer's instructions and the number of fluorescein-positive cells in the microscopic fields of each section was determined by fluorescence microscope.

### Immuno-histochemical staining for VEGF receptor

Paraffin-embedded tissue sections were treated with xylene to remove the paraffin, and then dehydrated in ethanol, and re-hydrated in PBS. Endogenous peroxidase activity was neutralized by incubating the sections for 20 min in 3% H2O2. After blocking the non-specific binding sites with 3% BSA and 5% normal goat serum, the section were incubated with primary antibodies for 1 h at room temperature. The primary antibodies were mouse monoclonal antibodies against VEGF R1 and R2 (Chemicon International, Inc. 1: 200 dilutions). Immuno-histochemical staining of VEGF R1 (Flt-1) and R2 (KDR) was done using standard techniques, with negative controls obtained by omitting the primary antibody.

### Statistical analysis

Statistical analysis was performed using the SPSS 9.0 for Windows (Statistical Package for Social Sciences, Inc., Chicago, IL) and analyzed using the Mann-Whitney test for non-parametric data. A *p *value < 0.05 was considered statistically significant.

## Results

### Morphometric measurements of airspace size

**Figure 1:**
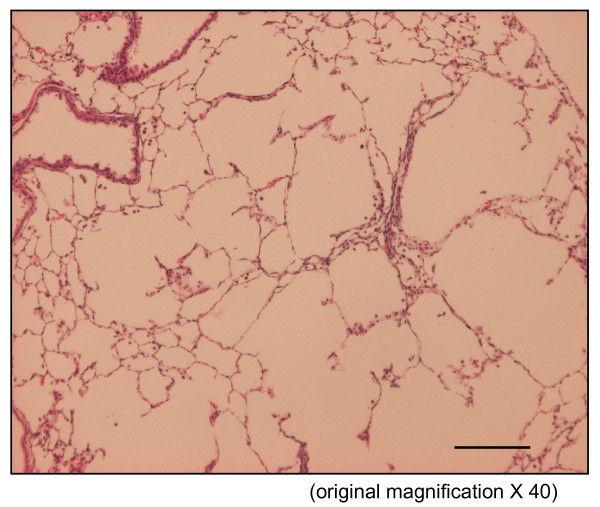
**Photo-micrograph of lung parenchyma after PPE or normal saline treatment - Wide type mice (PlGF +/+) treated with PPE for 4 weeks show alveolar wall destruction**.

**Figure 2:**
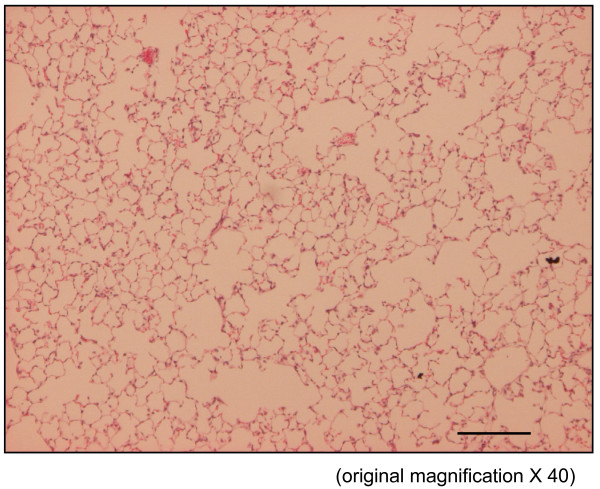
**Photo-micrograph of lung parenchyma after PPE or normal saline treatment - displays marked enlargement of airspace as compared to knock-out mice**.

**Figure 3:**
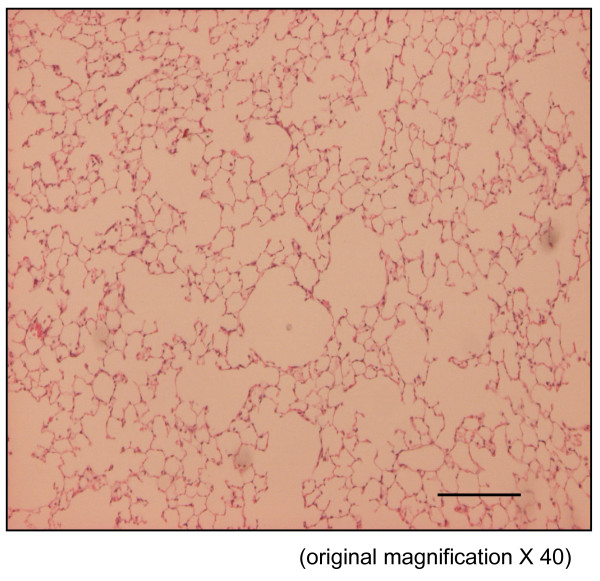
**Photo-micrograph of lung parenchyma after PPE or normal saline treatment - also displays marked enlargement of airspace as compared to the control group treated with normal saline**.

**Figure 4:**
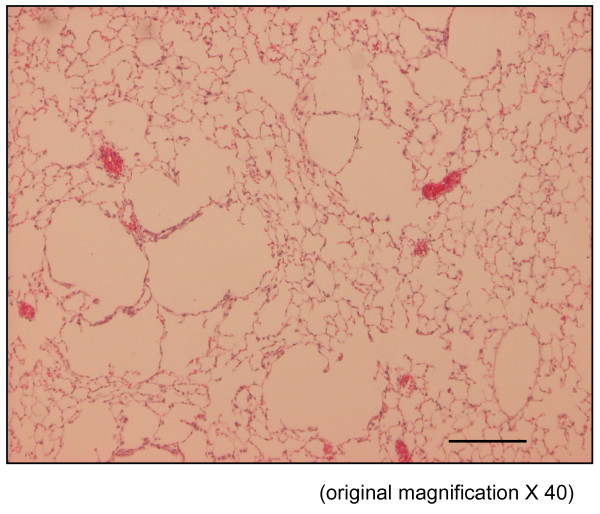
**The severity of emphysema is considerably less in panel (PlGF +/-, heterozygous type) (original magnification × 40; Bar = 100 μm)**. C: control.

The time course in developing emphysema after PPE instillation was examined in these various genotypes of mice. After 4 weeks of PPE treatment, there was marked alveolar enlargement with breaks in the alveolar walls compatible with destruction of the normal small airway structure in WT mice (Fig. 1). However, this was not present in PlGF KO mice (Fig. 2) and in saline control groups (Fig. 3). The degree of airspace enlargement was reduced in HE mice (Fig. 4).

**Figure 5:**
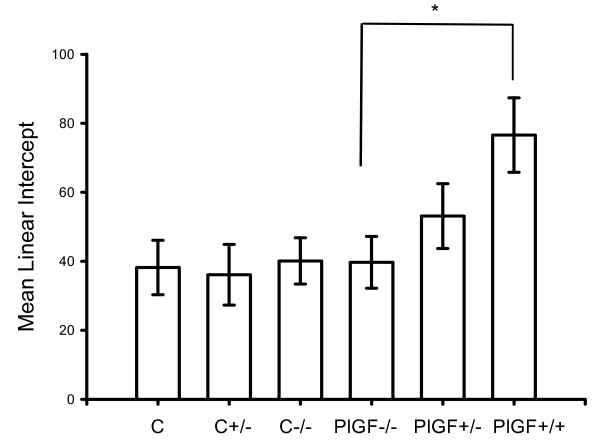
**Emphysema in PPE-treated lung was assessed by mean linear intercept (MLI)**. MLIs are significantly greater in the wild type and heterogeneous mice, compared to the PlGF KO mice or control groups. (*p < 0.05, C: wide type mice with saline; C+/-: heterozygous mice with saline; C-/-: homozygous deficiency mice with saline). An decrease in MLIs and the degree of emphysematous change correlate with KO mice when compared with Fig. 1B.

Upon morphologic quantification of the severity of emphysema by determining mean linear intercepts (MLI), the values of which were significantly greater in WT mice than in KO mice and controls (Fig. 5). Furthermore, the volume density of airspaces (V, _v(air, lung)_; %) was significantly higher in WT mice (87.2 ± 0.6%) treated with PPE for 4 weeks than the KO mice (71.8 ± 0.5%) (*p *< 0.01). PlGF KO mice had less degree in the development of PPE-induced emphysema. Besides, no significant MLI increase was detected in heterozygous and homozygous deficiency mice with saline treatment.

### Decreased MMP-9 and TNF-alpha expression in lungs of PlGF KO mice

**Figure 6:**
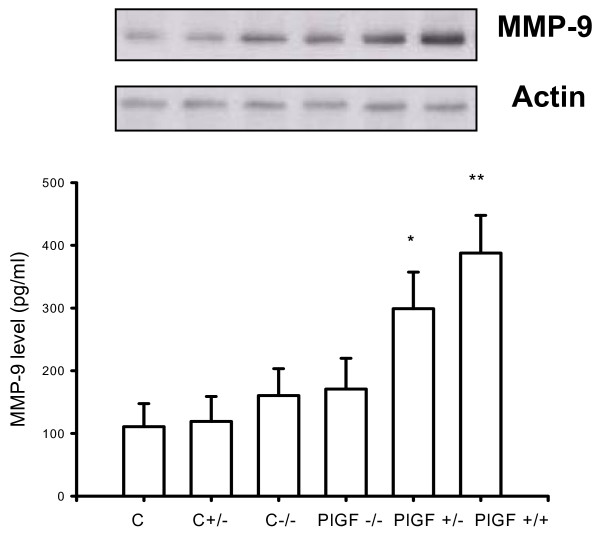
**ELISA and Western blot analysis show higher expression of MMP-9 in PlGF +/+ wild type mice, compared with PlGF -/- KO mice**.

**Figure 7:**
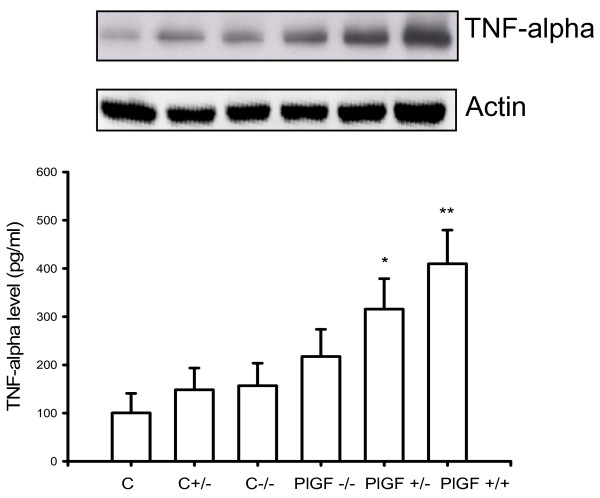
**ELISA and Western blot analysis show higher expression of TNF-α in PlGF +/+ wild type mice, compared with PlGF -/- KO mice**.

**Figure 8:**
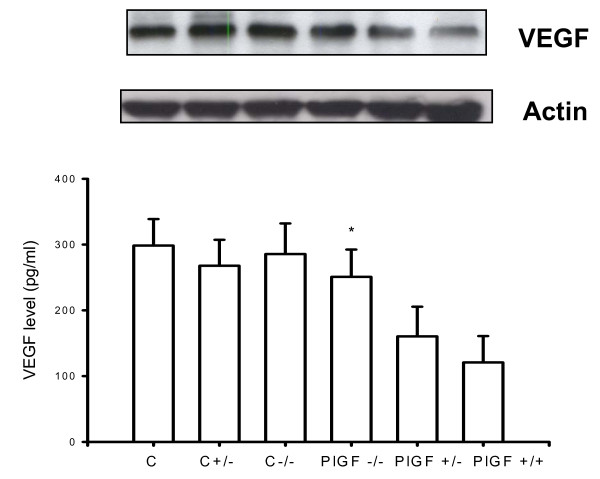
However, VEGF expression is higher in PlGF KO mice than in wild type mice (*p < 0.05; **p < 0.01, C: wide type mice with saline; C+/-: heterozygous mice with saline; C-/-: homozygous deficiency mice with saline).

To assess if inflammatory mediators were affected in PlGF KO mice, MMP-9 and TNF-alpha expression were analyzed after 4 weeks, as well as VEGF expression. The expressions of both MMP-9 and TNF-alpha were lower in the lungs of PlGF KO mice than in WT mice (Figs. 6 and 7). However, VEGF expression was higher in KO mice than in the WT mice (Fig. 8), which revealed decreased inflammatory reactions but increased vasculature in KO mice after PPE instillation.

### Decreased pulmonary septal cell death in lungs of PlGF KO mice

**Figure 9:**
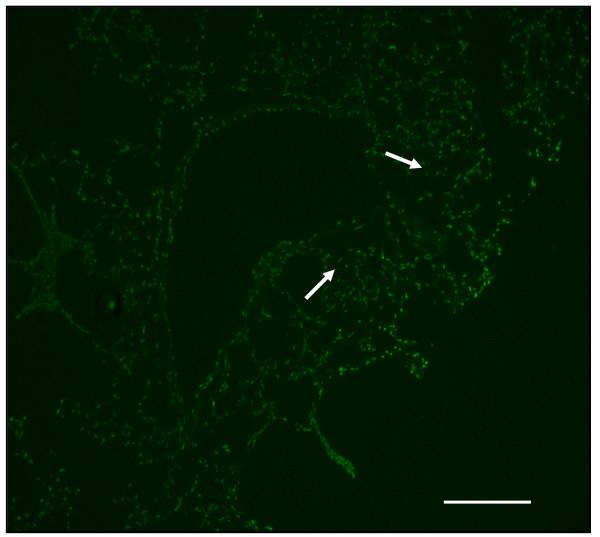
**Increased apoptotic cells in the alveolar septa of PlGF WT mice**.

**Figure 10:**
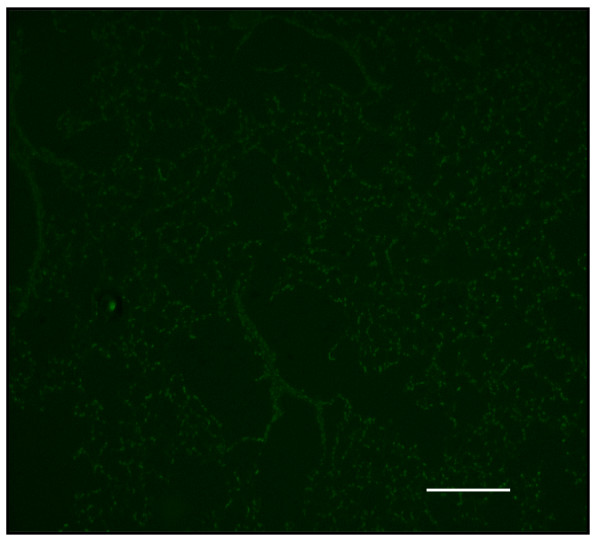
**There is increased terminal deoxynucleotidyl (TdT)-mediated dNTP nick end-labeling (TUNEL)-positive cells (fluorescent, arrows) after a 4-week PPE treatment (original magnification × 40, Bar = 100 μm) compared to KO mice**.

**Figure 11:**
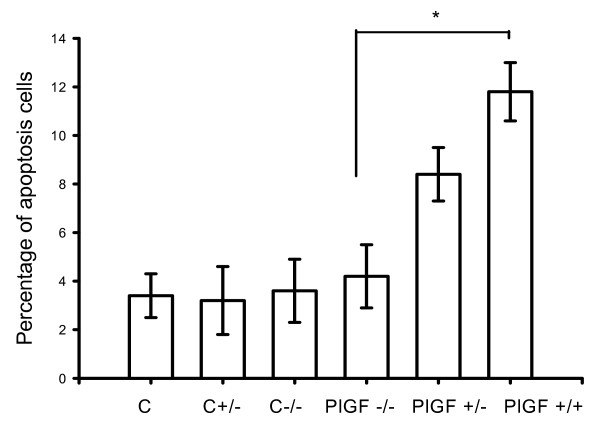
**TUNEL-positive cells are counted and represented in the graph**. There are significantly more TUNEL (+) cells in the emphysema lungs (PPE-treated WT mice) compared to the lungs of HE, KO, and control mice (p < 0.01, C: wide type mice with saline; C+/-: heterozygous mice with saline; C-/-: homozygous deficiency mice with saline). In PlGF KO mice, there is weekly PPE instillation followed by exogenous PlGF at a dose of 1 mg/kg via intra-tracheal route.

Assessment of apoptotic cells in the alveolar septa normalized by fluorescence microscopy from serial sections revealed an increase in TUNEL (+) cells in emphysematous lungs when compared to those of PlGF KO mice (Figs. 9 and 10). Quantification of the number of apoptotic cells in the alveolar septa normalized by the amount of nucleic acid extracted from serial sections revealed an increase in TUNEL (+) cells in WT emphysematous lungs when compared with the lungs of HE or KO mice (Fig. 11). There were significantly more TUNEL (+) cells in the WT (emphysema, 11.8 ± 1.2%) than in KO mice (4.2 ± 1.3%) (*p *< 0.01).

### Re-development emphysema after exogenous PlGF instillation in PPE-treated PlGF KO mice

**Figure 12:**
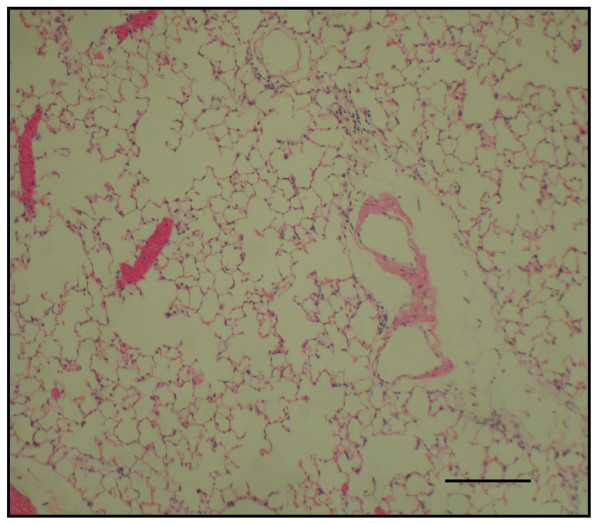
**KO mice treated with 4 weeks of PPE reveal no marked airspace enlargement**.

**Figure 13:**
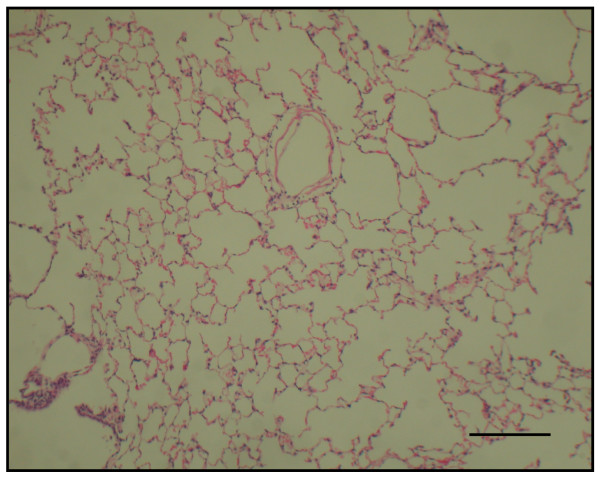
**Some emphysematous change is detected at 2 weeks of concomitant therapy**.

**Figure 14:**
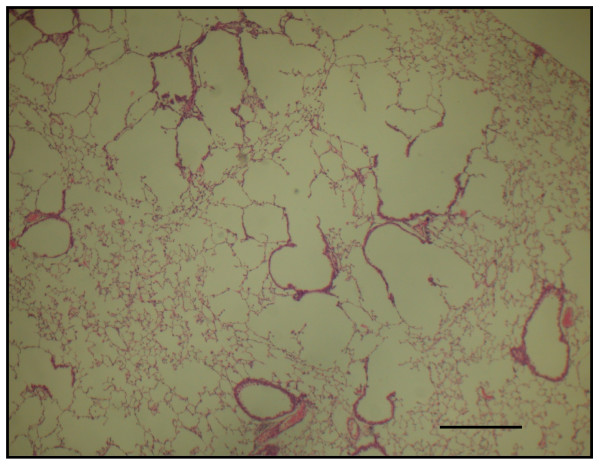
**The airspaces are significantly enlarged after 3 weeks of treatment**.

**Figure 15:**
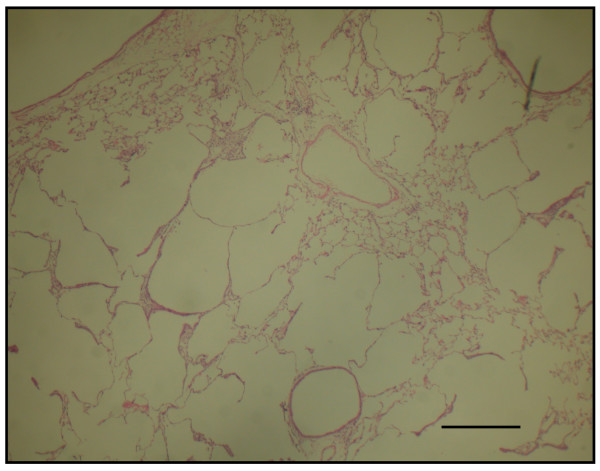
**The airspaces are markedly larger than before the 4-week treatment, and emphysema re-develops (original magnification × 40, Bar = 100 μm)**.

PlGF KO mice were given weekly PPE instillation followed by exogenous PlGF. Compared without exogenous PlGF therapy (Fig 12), emphysematous changes were detected within 2 weeks of concomitant therapy (Fig 13). Airspace enlargement became more significant after 3-4 weeks. (Figs. 14, 15) Emphysema re-developed after exogenous PlGF instillation in PPE-treated PlGF KO mice, which implied that PlGF contributed to the development of emphysema.

PPE instillation followed by administration of neutralizing anti-VEGFR1 antibody AF471 decreased the development of emphysema after 4 weeks. The MLIs values decreased by 36% in the AF471 treatment group compared to the controls (78 ± 17 vs. 60 ± 18, *p *= 0.06), which did not reach statistical significance.

### Decreased expression of immuno-histochemical staining for VEGF receptor

**Figure 16:**
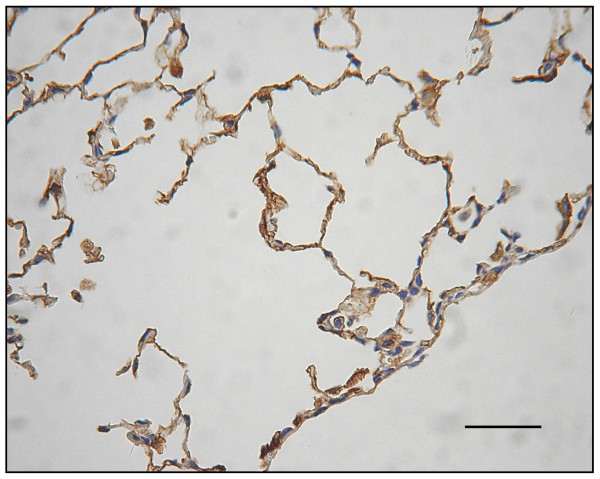
**Immuno-histochemical staining for VEGF R1 (Flt-1) and VEGF R2 (KDR)**. In Flt-1 expression, these are significantly decreased in emphysematous lungs.

**Figure 17:**
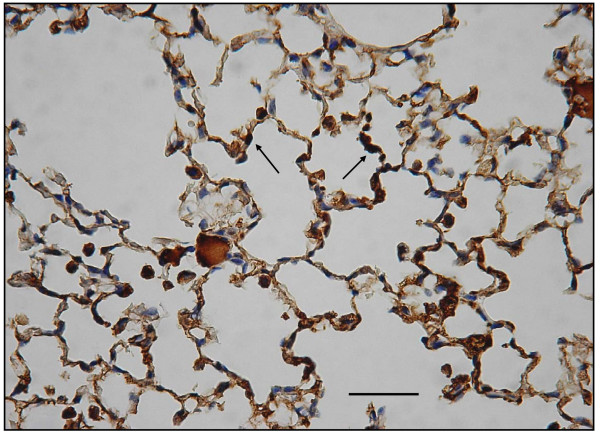
**Immuno-histochemical staining for VEGF R1 (Flt-1) and VEGF R2 (KDR) Displays Flt-1 expression comparison in controls (arrows)**.

**Figure 18:**
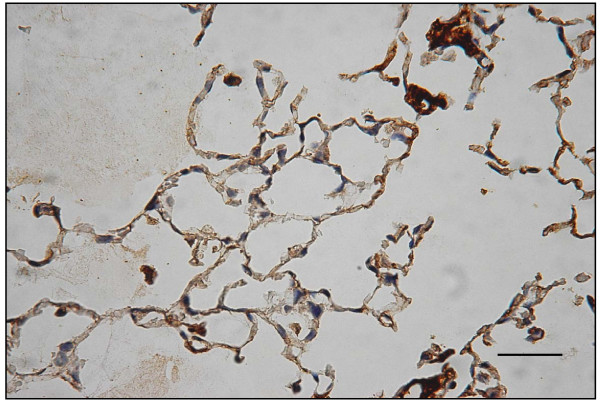
**In KDR expression, these are significantly decreased in emphysematous lungs**.

**Figure 19:**
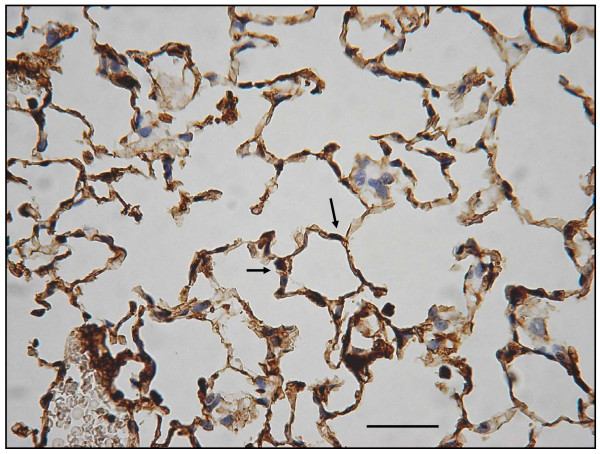
**Displays KDR expression comparison in controls (arrows)**. (original magnification × 200. Bar = 100 μm).

**Figure 20:**
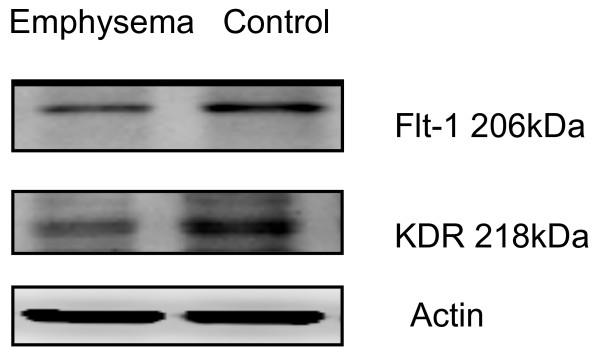
**Western blot analysis shows lower VEGFR1 and VEGFR2 expression in emphysematous tissues compared to controls**.

In the emphysematous tissues of WT mice treated with 4 week PPE, VEGF R1 (Flt-1) expression decreased as compared to the WT control mice with saline that had no emphysema (Figs. 16 and 17). Moreover, there was reduced VEGF R2 (KDR) expression in lungs with emphysematous changes (Figs. 18 and 19). Western blot analysis also confirmed the reduced expression of these receptors (Fig. 20).

## Discussion

Pulmonary emphysema, defined as abnormal airspace enlargement distal to the terminal bronchioles, is a major component of COPD. Although COPD occurs predominantly in smokers, the fact that only 15-20% of smokers develop pulmonary emphysema suggests an interaction of genetic, environmental, and other factors in causing emphysema [18-21]. The protease-anti-protease imbalance and oxidative stress theories related to inflammation are considered the key pathogenesis behind pulmonary emphysema. However, inflammation may not be the sole mechanism. Previously, studies reported the association of VEGF with COPD [8,9]. In addition, Tsao et al. have demonstrated that PlGF transgenic mice develop pathology similar to human pulmonary emphysema, [13] while humans with COPD show elevated PlGF levels in sera and BAL fluids [14]. Taken together, angiogenic growth factors, such as PlGF, may contribute to the development of emphysema.

The current study demonstrates that PlGF KO mice are protected from developing elastase-induced emphysema. It also shows lower apoptosis cell counts in PlGF KO mice that did not develop emphysema, when compared to WT mice that developed emphysema. Based on previous findings [14], persistent PlGF treatment, combined with TNF-α and IL-8, induces the down-regulation of VEGF in human bronchial epithelial cells, most likely through reduced number of viable cells and increased cell apoptosis. Tsao et al. have shown that PlGF inhibits the proliferation of MLE-15 cells (a mouse pulmonary type II epithelial cell line) in a dose-dependent manner and significantly promotes cell death [13]. Findings in cell culture studies are compatible with those from animals. Moreover, intra-tracheal instillation of exogenous PlGF in elastase-treated PlGF KO mice re-develops the emphysematous pattern. We thought that PlGF is essential in the pathogenesis of emphysema and is related to apoptosis.

Previously, it has been demonstrated that *in vitro *chronic stimulation of epithelial cells with PlGF and other cytokines induce cell death and apoptosis, which is similar to exposure to chronic irritants associated with *in vivo *lung parenchymal damage [14]. A VEGFR inhibitor in a concentration that mainly blocks VEGF R1 abolished this phenomenon [14]. In this study, VEGF receptors, including VEGF R1 and R2, have decreased expression in emphysematous tissues. It is speculated that inflammatory cytokines (i.e. TNF-α, IL-8) and PlGF-induced alveolar cell apoptosis reduce the expression of VEGF and down-regulate VEGFR. These result in fewer endothelial cells and thin, avascular alveolar septum that are compatible with Liebow's opinion [22].

Recent studies pointed out that the failure to maintain alveolar structure and lung apoptosis contributes to the development of emphysema [23,24]. The defective homeostasis of one or more cell types elicit emphysematous changes. For instance, when VEGF, which is abundant in the lungs, is neutralized in animal models, the result is an apoptosis-dependent enlargement of airspaces and structural changes similar to emphysema [25-27], not only by induction of apoptosis of type II pneumocytes but also by impaired production of surfactant [13,28]. An increasing number of data, from animal models, studies on human subjects, and cell culture experiments, supports an important role for apoptosis in the pathogenesis of emphysema. Thus, several disease mechanisms are involved in the process, including inflammation, proteinase-anti-proteinase imbalance, and oxidative stress. Apoptosis interacts with all of these pathways, adding to the complexity of the disease.

PlGF expression increases significantly in early gestation, peaks at around 26-30 weeks, and decreased as term approaches [29]. However, the biological function of PlGF after gestation and in adulthood remains unclear. Although the synergism between VEGF and PlGF contributes to angiogenesis and plasma extravasation in pathologic conditions such as ischemia or inflammation [15], it has been demonstrated that the bronchial epithelial cells can express PlGF and elevated levels of PlGF have harmful effects in COPD patients [14]. This animal study shows that PlGF KO mice are protected against emphysema. Since persistent and elevated PlGF levels may induce pulmonary cell damage, inhibiting PlGF offers opportunities for blocking the development of emphysema.

Several animal models of COPD development have been previously studied. Compared to the chronic smoke exposure model, the model of elastase-induced emphysema develops more acutely, even though it may have more limited clinical relevance, which included small airway disease (bronchiolitis), airflow limitation, COPD exacerbation, systemic inflammation and extrapulmonary manifestation. Some animal studies demonstrate that inhibiting VEGFRs causes alveolar wall or endothelial cell apoptosis, which is sufficient to cause emphysema. However, this does not lead to any accumulation of inflammatory cells [26,30] or VEGF [31]. In the PPE-instillation emphysema animal model, there is a substantial inflammatory response (MMP and TNF-α) accompanied by an increase in cellular apoptosis and down-regulation of VEGF levels, which are all relevant pathologic characteristics of COPD.

Based on the previous *in vitro *study, the chronic activation of epithelial cells with PlGF and other cytokines induces cell death and apoptosis, which can be abolished by a VEGF R1 inhibitor [14]. In this study, exogenous VEGF R1 blocker prevented emphysema in mice, with a trend of decreased level of airspace enlargement. However, the decrease expression of VEGF R1 and R2 in emphysema tissues had not been expected. Aside from receptor blockers, more studies should be performed to test whether apoptosis can be a therapeutic target to prevent emphysema.

In conclusion, this study demonstrates the hypothesis that blocking PlGF can prevent the development of PPE-induced emphysema in mice. The pathogenesis may be related to the apoptosis. A VEGF R1 blocker partially inhibited the action of exogenous PlGF and caused re-development of emphysema. Identifying the cellular and molecular mechanisms in the pathogenesis of emphysema and apoptosis should have important implications in developing new targets for therapeutic intervention of COPD.

## Competing interests

The authors declare that they have no competing interests.

## Authors' contributions

SLC carried out the animal studies, participating molecular biology experiments and drafted the manuscript. SJC carried out the apoptosis with TUNEL stain. PC and PNT participated in the source of the gene-deficient mice. SLC and HCW participated in the design of the study and performed the statistical analysis. CJY and PCY conceived of the study, and participated in its design and coordination and helped to draft the manuscript. All authors read and approved the final manuscript.
